# Polystyrene microplastics compromise intestinal health and reduce growth and slaughter performance of Chahua chicken No. 2

**DOI:** 10.3389/fmicb.2026.1954352

**Published:** 2026-09-30

**Authors:** Kun Wang, Yidan Ma, Shiyu Chen, Chen Liu, Guo Chen, Linna Duan, Le Xu, Xiannian Zi, Tengfei Dou, Qiuye Lin, Shunfen Zhang, Zhenhui Cao

**Affiliations:** 1 Faculty of Animal Science and Technology, Yunnan Agricultural University, Kunming, China; 2 Key Laboratory of Animal Nutrition and Feed Science of Yunnan Province, Kunming, China; 3 School of Ethnic Medicine, Yunnan Minzu University, Kunming, China

**Keywords:** Chahua chicken No. 2, growth performance, intestinal barrier, microbiome, microplastics

## Abstract

Microplastics are ubiquitous in livestock settings and raise increasing concern, yet studies on their effects on poultry intestinal health and gut microbiota, particularly in native Chinese breeds, are still scarce. Chahua chicken No. 2 were exposed to drinking water with 0, 0.05, 0.1, or 0.5 mg/d polystyrene microplastics (PS-MPs). At 0.5 mg/d, PS-MPs significantly reduced body weight, average daily gain, and feed intake at 42 and 120 days, while increasing feed conversion ratio and decreasing carcass traits (semi-eviscerated yield, eviscerated yield, breast and thigh muscle weights) at 42 days (*p* < 0.05). This dose also increased the microplastic-associated fluorescence signal in jejunum and ileum, reduced villus height and villus height/crypt depth ratio, and deepened crypts. It downregulated genes associated with intestinal barrier integrity (*ZO-1*, *Occludin*, *Claudin-1*), upregulated inflammatory cytokines (*IL-6*, *IL-8*, *IL-1β*, *TNF-α*, *MIP-1β*), and suppressed genes involved in glucose (*SLC5A1* and *SLC2A2*), lipid (*SLC27A4*, *APOA1*, *APOB*, *MTTP*, *CD36*, *FABP2*), and amino acid (*SLC15A1*, *SLC7A1*, *SLC7A9*, *SLC3A1*, *SLC6A19*) transport (*p* < 0.05). 16S rRNA gene sequencing analysis revealed that 0.5 mg/d PS-MPs exposure induced intestinal microbiota dysbiosis in Chahua chicken No. 2, characterized by an increased abundance of *norank_o_Clostridia_UCG_014, Mediterraneibacter, Olsenella*, and *Faecalitalea* (*p* < 0.05). Overall, these findings indicate that PS-MP exposure is associated with alterations in growth performance, intestinal morphology, barrier-related gene expression, inflammatory responses, nutrient transporter expression, and gut microbiota in Chahua chicken No. 2.

## Graphical abstract

**Figure:**
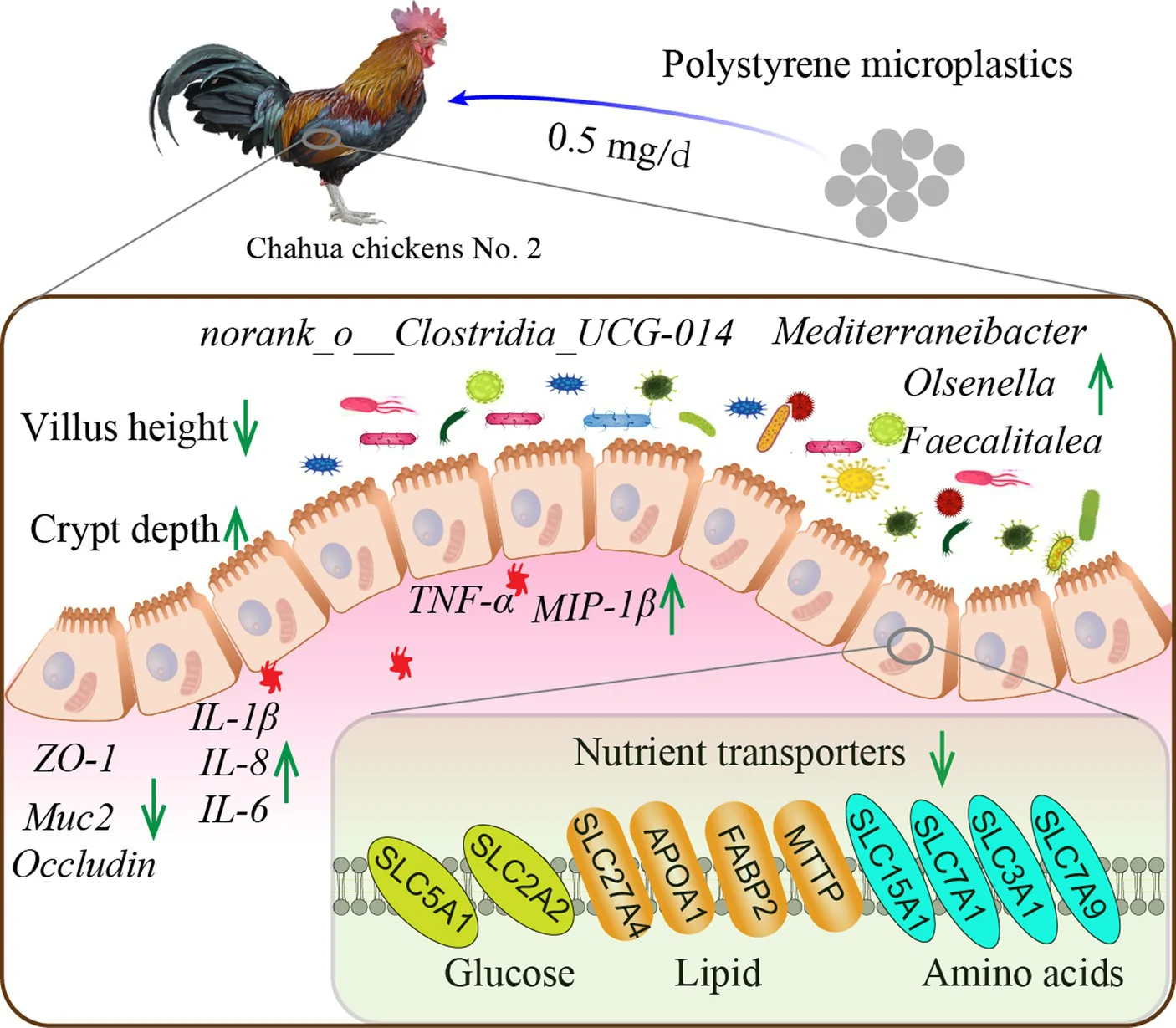

## 1 Introduction

With the widespread production and extensive use of plastic products, microplastics (MPs) pollution has emerged as a global environmental concern (Goswami et al., 2024; Thacharodi et al., 2024). Microplastics are now distributed across freshwater, terrestrial, and marine ecosystems and have been detected in a wide range of organisms (Li et al., 2025). Among various types of microplastics, polystyrene microplastics (PS-MPs) are considered to pose relatively high exposure risks to both animals and humans due to their widespread sources and high environmental detection frequency (Yin et al., 2023; Kwon et al., 2022). A growing body of evidence has demonstrated that MPs can accumulate in multiple species and exert a variety of adverse biological effects (Zhang et al., 2024; Ali-Hassanzadeh et al., 2025). For instance, MPs exposure has been associated with metabolic disorders, neurotoxicity, and an increased risk of cancer, thereby posing potential threats to human health (Sangkham et al., 2022; Rahman et al., 2021). In poultry, MPs exposure has been reported to disrupt energy and fatty acid metabolism and to trigger immune and inflammatory responses in the liver (Xu et al., 2025).

Poultry production systems are especially vulnerable to MPs contamination, as MPs can enter through feed, drinking water, and bedding materials, posing potential risks to animal health and food safety (Bristi and Islam, 2026; Bouwmeester et al., 2015). Previous studies have detected MPs residues in the intestine, liver, and skeletal muscle of chickens (Chen et al., 2023). Chahua chicken No. 2 is a breed developed from the native Chahua chicken in Southwest China, widely raised due to its strong environmental adaptability and superior meat quality (Liu et al., 2022; Alsoufi et al., 2022). However, the effects of microplastics on Chinese indigenous chickens, including Chahua chicken No. 2, have not been reported yet.

The intestine was a primary target organ for MPs toxicity, serving as the central site for nutrient digestion, absorption, and immune defense, while also hosting a complex microbial ecosystem critical for metabolic and immune homeostasis (Chartres et al., 2024; Liang et al., 2021). Although MPs-induced intestinal dysfunction and microbiota dysbiosis have been documented in fish and mammals (Liang et al., 2024; Li et al., 2024b), the avian gastrointestinal tract exhibits distinct anatomical and physiological characteristics, suggesting that MPs toxicity mechanisms in chickens may differ. Research specifically addressing MPs-induced alterations in intestinal morphology, barrier-related responses, nutrient transporter expression, and gut microbiota in poultry remains limited to date. We hypothesized that microplastic exposure would adversely affect intestinal health and growth performance in Chahua chicken No. 2 in a dose-dependent manner.

Therefore, this study aimed to systematically evaluate the effects of different doses of microplastics on growth performance, intestinal morphology, intestinal barrier-related gene expression and inflammatory responses, nutrient transporter-related gene expression, and gut microbial communities in Chahua chicken No. 2. This work provides insights into the intestinal responses to microplastic exposure in the Chahua chicken No. 2 breed and contributes to the understanding of the potential biological effects of MPs contamination in poultry.

## 2 Materials and methods

### 2.1 Chickens and experiment design

Chahua chicken No. 2 were provided by Yunnan Agricultural University practice chicken farm. A total of 120 one-day-old Chahua chicken No. 2 were used in this experiment and randomly allocated to 4 groups, each with 3 independent replicate cages, and each replicate contained 10 chickens. The chicks were randomly selected for allocation to the treatment groups while maintaining an equal sex ratio among groups. The experimental design included three independent replicate cages per treatment group to provide cage-level biological replication while limiting the total number of animals used. All birds were housed in three-tier step-type metal cages (10 birds per cage, stocking density approximately 20 birds/m^2^) with ad libitum access to feed and water. During the brooding period (days 1–21), room temperature was maintained at 32–35 °C, then reduced by 2–3 °C per week until reaching 22 °C; relative humidity was kept at 60–65%. The lighting schedule was 23 L:1D for days 1–3, gradually changed to 16 L:8D by day 42, and natural lighting thereafter. Routine vaccination against Newcastle disease and infectious bronchitis was administered according to the standard commercial protocol. The chickens were administered polystyrene microplastics (PS-MPs) via drinking water at doses of 0 mg/day (CON), 0.05 mg/day (0.05 PS-MPs), 0.10 mg/day (0.1 PS-MPs), and 0.50 mg/day (0.5 PS-MPs), respectively. These doses represent the total nominal amounts of PS-MPs provided to each treatment group during days 1–42. Based on 30 chickens per treatment group, the corresponding theoretical average exposures were 0, 0.00167, 0.00333, and 0.0167 mg/bird/day, respectively. The PS-MPs were spherical particles with a diameter of 5 μm and were labeled with green fluorescence (excitation/emission wavelengths: 488/520 nm). The particles were purchased from Jiangsu Zhichuan Technology Co., Ltd. (China). According to the manufacturer, the microspheres consisted of pure polystyrene (>99% purity) and had a uniform particle-size distribution. The required amount of PS-MPs was freshly dispersed in approximately 200 mL of drinking water each morning for each treatment group and equally distributed among the three replicate cages, with approximately 66.7 mL provided to each cage in a separate drinking vessel. The suspension was thoroughly mixed before administration and mixed again immediately before use to minimize particle settling. The suspension was consumed before noon, after which regular drinking water was provided for the remainder of the day. The same volume of drinking water without PS-MPs was provided to the CON group. No additional surfactants, preservatives, or other dispersing agents were added, and the exposure suspensions were freshly prepared each morning and were not stored overnight. The experimental diets were formulated to meet the nutrient requirements of poultry as recommended by the National Research Council (NRC, 1994) (Table 1). Daily feed intake was recorded at the replicate level, and feed conversion ratio (FCR) was calculated accordingly. For feed intake, average daily feed intake (ADFI), FCR, and other cage-level performance parameters, the replicate was considered the experimental unit. At 42 days of age, three birds were randomly selected from each replicate pen, and one additional bird was randomly selected from the remaining birds in each treatment group, resulting in 10 birds per group for slaughter and carcass performance measurements. Samples of the jejunum and ileum were collected, snap-frozen in liquid nitrogen, and stored at −80 °C for further analysis. For gut morphology, qRT-PCR, and microbiome analyses, the selected animals were derived from the three independent experimental replicates within each treatment. Histomorphometric measurements were performed by an investigator blinded to treatment allocation. The remaining chickens continued to receive the corresponding treatment until 120 days of age, with the amount of PS-MPs provided adjusted according to the number of chickens remaining in each replicate cage to maintain the same theoretical average exposure per bird as during days 1–42. Body weight and slaughter performance were measured at 120 days of age.

**Table 1:** Experimental diet composition and nutrient level (air-dry-basis).

Item	1–42 days diet (%)	43–120 days diet (%)
Corn	58.93	55.00
Soybean meal	25.93	30.00
Corn gluten meal	6.36	2.51
Wheat Bran	–	4.47
Soybean oil	3.63	3.52
Limestone	2.00	0.90
Dicalcium phosphate	1.50	2.00
Lysine	0.15	0.07
Methionine	0.25	0.29
Salt	0.26	0.25
Premix^1^	1.00	1.00
Total	100.00	100.00
Nutrient levels^2^		
Metabolizable energy (MJ/kg)	12.75	12.56
Crude protein	21.98	20.03
Methionine	0.52	0.61
Lysine	1.20	1.05
Calcium	0.96	0.96
Total phosphorus	0.85	0.66
Available phosphorus	0.61	0.47

^1^The premix provides per kg of diet: vitamin A, 4000 IU; vitamin D3, 1,200 IU; vitamin E, 15 IU; vitamin K, 0.5 mg; vitamin B1, 2.8 mg; vitamin B2, 4.0 mg; vitamin B3, 35 mg; vitamin B5, 20 mg; vitamin B6, 3.5 mg; vitamin B12, 0.01 mg; biotin, 0.2 mg; folic acid, 0.6 mg; choline chloride, 1,000 mg; Cu, 9 mg; Fe, 90 mg; Zn, 90 mg; Mn,80 mg; I, 0.25 mg; Se, 0.15 mg. ^2^Crude protein, calcium, and total phosphorus were measured values, and the others were calculated values.

### 2.2 Hematoxylin and eosin (H&E)

Fresh jejunum and ileum tissues were collected and fixed in Carnoy’s solution for more than 24 h, followed by dehydration, paraffin embedding, and sectioning. For histomorphometric analysis, six chickens per group (randomly selected from the three replicate pens) were examined. From each chicken, one tissue section was prepared, and six well-oriented villi (with intact epithelium and clear crypt–villus junctions, sectioned perpendicularly to the muscularis mucosa) were randomly selected per section for hematoxylin and eosin (H&E) staining. Villus height and crypt depth were measured under an Olympus BX53 light microscope (Olympus Corporation, Tokyo, Japan) at ×10 magnification, and images were captured and analyzed using ImageJ software (National Institutes of Health, Bethesda, MD, United States). All measurements were performed by an investigator blinded to treatment allocation. For each chicken, the six individual measurements of villus height and crypt depth were averaged to generate a single biological replicate value.

### 2.3 16S rRNA gene sequencing analysis

Ileal contents were collected for 16S rRNA gene sequencing analysis. Microbial DNA was extracted using the E. Z. N. A.^®^ soil DNA Kit (Omega Bio-tek, Norcross, GA, United States) according to manufacturer’s instructions. The V3–V4 hypervariable region of the bacterial 16S rRNA gene was amplified using the primer pair 338F (5’-ACTCCTACGGGAGGCAGCAG-3′) and 806R (5’-GGACTACHVGGGTWTCTAAT-3′) on a T100 Thermal Cycler (BIO-RAD, United States). The PCR product was extracted from 2% agarose gel and purified using the PCR Clean-Up Kit (YuHua, Shanghai, China) following the manufacturer’s protocol, and quantified using a Qubit 4.0 fluorometer (Thermo Fisher Scientific, United States). Purified amplicons were pooled in equimolar amounts and subjected to paired-end sequencing on the Illumina Nextseq2000 platform (Illumina, San Diego, USA) according to the standard protocols by Majorbio Bio-Pharm Technology Co. Ltd. (Shanghai, China). The raw sequencing reads were deposited into the NCBI Sequence Read Archive (PRJNA1441598) database.

Data preprocessing and subsequent analyses were performed using the QIIME2 pipeline (version 2023.5) on the Majorbio I-Sanger cloud platform (www.i-sanger.com, Majorbio, Shanghai, China). Raw paired-end reads were generated using a 300-bp paired-end (PE300) sequencing configuration. The raw reads were quality-filtered using fastp (v0.19.6) and merged using FLASH (v1.2.7). The expected insert size of the 338F_806R amplicon was approximately 468 bp. The resulting merged sequences were subsequently denoised using the DADA2 plugin in QIIME 2, with the maximum expected error (maxEE) set to 2.0. After quality control and chimera removal, high-quality amplicon sequence variants (ASVs) were generated. The sequencing depth per sample ranged from 40,957 to 82,590 optimized sequences. To eliminate bias caused by differences in sequencing depth across samples, all samples were rarefied to an even sequencing depth (the minimum library size of 40,957 sequences per sample) using the built-in rarefaction pipeline of the Majorbio I-Sanger cloud platform.^1^ Rarefaction curves were generated, and Good’s coverage was calculated for each sample to verify the adequacy of sequencing depth; all coverage values exceeded 0.98, and the rarefaction curves approached saturation, indicating sufficient sequencing coverage for downstream analyses. Taxonomic classification of ASVs was performed using the SILVA reference database (version 138.2) with a confidence threshold of 0.7. Based on this rarefied ASV abundance matrix, relative abundance was calculated, and the following diversity and differential abundance analyses were conducted. For alpha diversity, the Ace, Chao, and Sobs indices were calculated. Differences among the four treatment groups were assessed using the Kruskal–Wallis test, followed by Dunn’s multiple comparisons test for pairwise comparisons when appropriate. For beta diversity, principal coordinate analysis (PCoA) and non-metric multidimensional scaling (NMDS) were applied based on the Bray-Curtis distance matrix calculated from the rarefied data, and statistical significance among groups was tested using analysis of similarities (ANOSIM) with 999 permutations. Correlations between the expression levels of genes and the abundance of gut microbiota were evaluated using Spearman’s rank correlation coefficient, and *p*-values were adjusted for multiple comparisons using the Benjamini–Hochberg false discovery rate (FDR) method.

### 2.4 Quantitative real-time polymerase chain reaction (qRT-PCR)

Total RNA was extracted from the jejunum and ileum using the phenol-chloroform method. RNA concentration was measured with a NanoDrop 2000 spectrophotometer (NanoDrop Technologies, Wilmington, DE, United States) and diluted to an appropriate concentration. Subsequently, cDNA was synthesized from the RNA using the PrimeScript™ RT Reagent Kit (Takara, Shiga, Japan). Gene expression levels were assessed by quantitative real-time PCR (qRT-PCR) using SYBR^®^ Premix Ex Taq™ (Takara), with *β*-actin serving as the internal reference gene. The primer sequences used for the analyses are listed in Table 2.

**Table 2:** List for qRT-PCR primers of chickens.

Gene number	Gene name	Primer (5^,^→3^,^)
NM_205518.2	*ACTB*	F: CGGACTGTTACCAACACCCA R: TCCTGAGTCAAGCGCCAAAA
XM_040706827.2	*ZO-1*	F: TCTTCCTCCTCCCGCTTCTTCAC R: AGAGATGGTGGTGTAGGCAGTGG
NM_001013611.2	*Claudin-1*	F: GACCAGGTGAAGAAGATGCGGATG R: CGAGCCACTCTGTTGCCATACC
NM_205128.1	*Occludin*	F: CTGCTCTGCCTCATCTGCTTCTTC R: CCATCCGCCACGTTCTTCACC
JX284122.1	*MUC-2*	F: CTGATTGTCACTCACGCCTTAATC R: GCCGGCCACCTGCAT
S40610.1	*IgA*	F: ACCACGGCTCTGACTGTACC R: CGATGGTCTCCTTCACATCA
NM_204628.2	*IL-6*	F: AACAACCTCAACCTGCCCAA R: AGGTCTGAAAGGCGAACAGG
NM_205498.2	*IL-8*	F: CCTCCTCCTGGTTTCAGCTG R: TGGCGTCAGCTTCACATCTT
NM_204524.2	*IL-1β*	F: CCTCCAGCCAGAAAGTGAGG R: TTGTAGCCCTTGATGCCCAG
NM_204267.2	*TNF-α*	F: ATCCTCACCCCTACCCTGTC R: TGTTGGCATAGGCTGTCCTG
NM_001030360.3	*MIP-1β*	F: TCTGCTACCAGACCTCTGCT R: GCCCTTCCTGGTGATGAACA
NM_001293240.2	*SLC5A1*	F: TGTGGGCATAGCAGGAACAG R: GCTTCCTCAGATACTCCGGC
NM_207178.2	*SLC2A2*	F: ATCCTCATCAGCCAGGTCCT R: TAGAGATAGCGGGGGCTCTC
XM_015279553.4	*SLC27A4*	F: AGGCCATCCCTGCTCTTTTC R: TGTTCTTCTCCCTCACGTGC
NM_001030731.1	*CD36*	F: CTCAACCTCGCTGTTGTTGC R: GGTCCAAGGGAAAGGGAACC
NM_001007923.2	*FABP2*	F: GCAATGGGCGTGAATGTGAT R: AAAGTTCAGTCCCGTCAGCC
NM_204192.4	*FABP1*	F: CTGCTGACAGGAGAGAAGGC R: TGGTGTCTCCGTTGAGTTCG
NM_205525.5	*APOA1*	F: CGCATTCGGGATATGGTGGA R: GTCAAGCTGTTTGCCCACAG
NM_001044633.2	*APOB*	F: GCCCAACATTTGCTGACGTT R: AGAAAGCCCAAGATCCCAGC
NM_001109784.3	*MTTP*	F: TGAAGGACATGCGTGCTCAT R: GTCTAGGCCGTACGTGGATG
AY029615.1	*SLC15A1*	F: AGTTCCCGCTGCCCTCATGG R: TTGCTGCGATGCCGAAACCTG
XM_004935370.5	*SLC3A1*	F: GACAAGGAGCTGAAGGGCTT R: CTTGGCTGCTGGTGTCAGTA
XM_419056.8	*SLC6A19*	F: GCTAACCCAGTGACATGGCT R: AGTCGCTGCGTAGATGGATG
NM_001145490.2	*SLC7A1*	F: TCTATGTACTGGCTGGGGCT R: AGGAACACGAGCACCGAATT
XM_046937871.1	*SLC7A7*	F: CACAAGCGAAAAACTGCGGA R: ACATTCCACCAAGAGCCCAG
NM_001199133.2	*SLC7A9*	F: CAGAACTCCTGCAGTCCCAG R: ACATACCCCATTGGCAGAGC

### 2.5 Distribution of microplastic-associated fluorescence in the chicken intestine

Jejunum and ileum tissues were collected from 42-day-old Chahua chicken No. 2. Intestinal contents were carefully removed, and the luminal surface was gently rinsed with pre-cooled phosphate-buffered saline (PBS). The tissues were immediately fixed in 4% paraformaldehyde fixative for 24 h at room temperature. After fixation, the target intestinal segments were trimmed flat, placed into embedding cassettes, dehydrated through graded ethanol, cleared in xylene, and finally infiltrated with paraffin at 65 °C. After infiltration, the tissues were oriented flat in embedding molds, filled with paraffin, and cooled and solidified on a −20 °C cold plate. The wax blocks were trimmed and set aside for sectioning. The trimmed wax blocks were sectioned at 4 μm thickness using a microtome for serial sections. The sections were then deparaffinized in an environmentally friendly dewaxing solution and rehydrated through absolute ethanol to distilled water. Subsequently, DAPI staining solution was applied and incubated for 8 min at room temperature in the dark to label cell nuclei. After washing three times with PBS, the slides were mounted with anti-fade mounting medium and examined under a fluorescence microscope for image acquisition. Fluorescence imaging was performed in multi-channel mode: DAPI nuclear staining used an excitation/emission of 330–380 nm/420 nm; fluorescently labeled microplastics (green-fluorescent PS-MPs) were detected using 488 nm excitation and 520 nm emission (FITC channel). All images were acquired under identical microscope settings. For each chicken, three random fields were selected from both the jejunum and ileum, avoiding tissue folds, damaged areas, and luminal debris. Regions of interest (ROIs) were consistently defined to cover the intestinal tissue area. Mean fluorescence intensity within each ROI was quantified using image analysis software, and the fluorescence intensity values were used to indicate the PS-MP-associated fluorescence signal and distribution of microplastics in the intestinal tissues.

### 2.6 Statistical analysis

Linear mixed-effects models were used as the primary analysis for outcomes measured at the individual-bird level, including slaughter performance, intestinal morphology, and gene expression. PS-MP treatment and sex were included as fixed effects, and replicate cage was included as a random effect to account for the non-independence of birds within the same cage. The models were fitted using the lme4 package, and estimated marginal means were obtained using the emmeans package. Pairwise comparisons among treatment groups were performed using Tukey’s adjustment for multiple comparisons. For cage-level outcomes, including body weight, ADG, ADFI, and FCR, the replicate cage was treated as the experimental unit, and analyses were performed using the replicate-level data. Thus, individual birds within the same cage were not treated as independent experimental replicates.

## 3 Results

### 3.1 Growth performance and slaughter performance of Chahua chicken No. 2 at 42 days of age

The effects of PS-MPs on the growth performance of Chahua chicken No. 2 were shown in Table 3. The body weight, ADG, and ADFI of Chahua chicken No. 2 at 42 days of age were significantly reduced by feeding 0.5 mg/d PS-MPs compared with the CON group (*p* < 0.05), whereas no significant differences were observed in the 0.05 and 0.1 mg/d PS-MPs treatment group. Furthermore, feeding 0.5 mg/d PS-MPs significantly increased the FCR of Chahua chicken No. 2 compared with the CON group and the 0.05 and 0.1 mg/d PS-MPs groups (*p* < 0.05).

**Table 3:** Effects of microplastics on the growth and slaughter performance of Chahua chicken No. 2 at 42 and 120 days of age.

Items	Group				*p* value
	CON	0.05 PS-MPs	0.1 PS-MPs	0.5 PS-MPs	
Day 1 BW (g)	46.86 ± 0.38	46.84 ± 0.67	46.82 ± 0.53	46.61 ± 0.93	0.901
Day 42					
BW (g)	618.67 ± 14.73^ab^	643.33 ± 19.49^a^	606.50 ± 28.03^b^	554.00 ± 23.67^c^	<0.001
ADG (g/d)	13.61 ± 0.40^ab^	14.20 ± 0.05^a^	13.33 ± 0.55^b^	11.68 ± 1.00^c^	<0.001
ADFI (g/d)	36.14 ± 0.63^ab^	36.55 ± 0.37^a^	35.25 ± 0.43^b^	35.16 ± 0.47^b^	0.020
FCR	2.66 ± 0.13^b^	2.57 ± 0.03^b^	2.68 ± 0.06^b^	3.02 ± 0.23^a^	0.016
Semi-eviscerated yield (%)	74.20 ± 5.00^a^	76.40 ± 6.66^a^	74.62 ± 5.00^a^	66.20 ± 4.96^b^	0.002
Eviscerated yield (%)	61.80 ± 5.20^a^	63.95 ± 6.25^a^	62.25 ± 3.32^a^	55.44 ± 4.26^b^	0.004
Breast muscle weight (g)	53.25 ± 8.10^a^	56.53 ± 8.78^a^	53.41 ± 5.48^a^	43.94 ± 6.94^b^	0.007
Thigh muscle weight (g)	71.86 ± 7.44^a^	76.06 ± 6.84^a^	72.65 ± 4.72^a^	57.18 ± 5.65^b^	<0.001
Breast muscle yield (%)	14.22 ± 1.99	14.35 ± 3.45	13.81 ± 1.89	13.70 ± 2.35	0.929
Thigh muscle yield (%)	19.24 ± 2.22	19.08 ± 1.85	18.77 ± 2.03	17.79 ± 1.47	0.346
Day 120					
BW (g)	2005.00 ± 108.95^a^	2051.67 ± 234.22^a^	1998.33 ± 49.97^a^	1766.67 ± 124.85^b^	0.018
ADG (g/d)	18.49 ± 0.25^a^	17.12 ± 1.39^ab^	15.58 ± 0.97^ab^	14.51 ± 1.22^b^	0.018
ADFI (g/d)	55.86 ± 0.66^ab^	56.12 ± 0.78^a^	53.86 ± 0.55^b^	51.68 ± 0.98^c^	<0.001
FCR	2.98 ± 0.05	3.29 ± 0.30	3.47 ± 0.24	3.57 ± 0.25	0.058
Semi-eviscerated yield (%)	80.82 ± 4.59	80.54 ± 3.20	79.74 ± 2.43	78.31 ± 6.93	0.786
Eviscerated yield (%)	66.27 ± 4.22	65.52 ± 3.76	65.33 ± 3.76	67.19 ± 5.60	0.875
Breast muscle weight (g)	240.11 ± 30.79^a^	215.66 ± 28.57^ab^	189.35 ± 14.50^b^	186.07 ± 20.78^b^	0.004
Thigh muscle weight (g)	285.78 ± 42.96^a^	256.71 ± 31.94^ab^	219.85 ± 12.99^b^	213.95 ± 28.23^b^	0.002
Breast muscle yield (%)	16.11 ± 2.40	15.79 ± 1.61	15.39 ± 2.66	15.48 ± 1.98	0.940
Thigh muscle yield (%)	19.06 ± 2.22	18.84 ± 2.12	17.82 ± 2.48	17.78 ± 2.38	0.655
Abdominal fat percentage (%)	5.17 ± 2.30	2.94 ± 1.86	5.30 ± 1.76	4.61 ± 2.00	0.128
Liver index	2.06 ± 0.40	2.02 ± 0.75	2.76 ± 0.69	1.83 ± 0.57	0.078
Spleen index	0.14 ± 0.05	0.19 ± 0.16	0.20 ± 0.04	0.15 ± 0.03	0.594
Thymus index	0.08 ± 0.07	0.09 ± 0.05	0.07 ± 0.05	0.06 ± 0.05	0.656

BW, body weight; ADG, average daily gain; ADFI, average daily feed intake; FCR, feed conversion ratio. Data are expressed as the mean ± SD. SD was calculated separately within each group. Within the same row, different lowercase letters indicate significant differences (*p* ≤ 0.05), while the same letters indicate no significant differences (*p* > 0.05).

The effects of PS-MPs on the slaughter performance of Chahua chicken No. 2 were shown in Table 3. The results showed that, compared with the CON group, 0.5 mg/d PS-MPs supplementation reduced the eviscerated yield, semi-eviscerated yield, breast muscle weight, and thigh muscle weight of Chahua chicken No. 2 (*p* < 0.05). In contrast, supplementation with 0.05 mg/d and 0.1 mg/d PS-MPs had no significant effects on these parameters (*p* > 0.05).

### 3.2 Growth and slaughter performance of Chahua chicken No. 2 at 120 days of age

The effects of PS-MPs on the growth performance of Chahua chicken No. 2 at 120 days old were shown in Table 3. The BW, ADG, and ADFI of Chahua chicken No. 2 at 120 days old in 0.5 PS-MPs group were significantly lower than CON group (*p* < 0.05), and the FCR has a tendency to be higher than that in CON group (*p* = 0.058). At 120 days of age, the breast muscle weight and thigh muscle weight of Chahua chicken No. 2 in the 0.1 PS-MPs and 0.5 PS-MPs groups was significantly lower than that of the control group (*p* < 0.05).

### 3.3 Distribution of microplastic-associated fluorescence in the chicken intestine

To visualize the presence of PS-MPs in chicken intestines, fluorescence microscopy was employed to detect green fluorescent signals associated with the particles (Figure 1A). The results showed that the green fluorescence intensity in the intestines of Chahua chicken No. 2 increased with higher exposure doses. Specifically, in the 0.5 PS-MPs group, the relative fluorescence intensity in both the jejunum and ileum was significantly higher than that in the CON group (*p* < 0.05, Figure 1B), indicating a dose-dependent distribution pattern of microplastic-associated signals.

**Figure 1:**
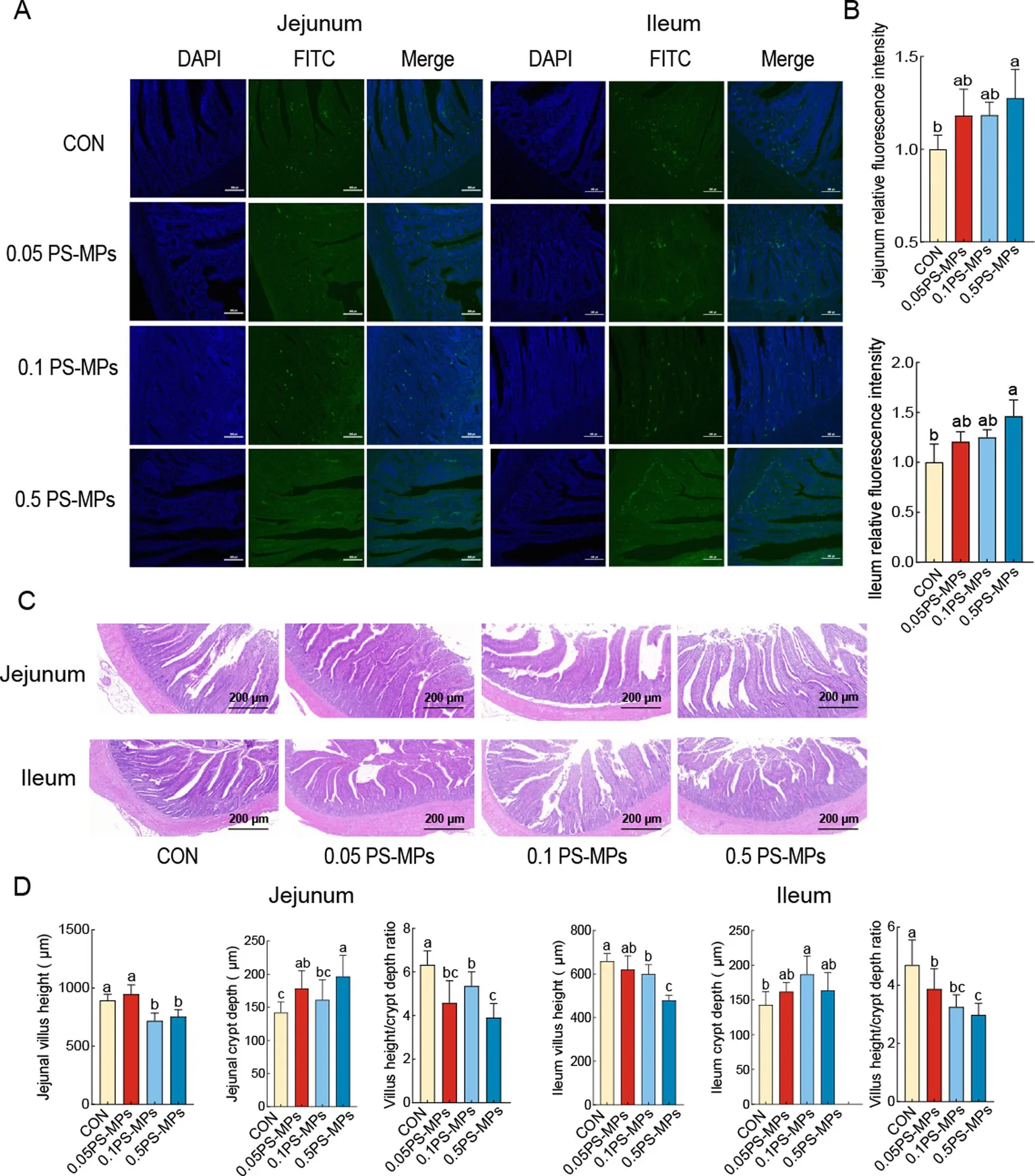
Distribution of microplastic-associated fluorescence in the intestine and intestinal morphology. **(A)** Green fluorescence of PS-MPs in the jejunum and ileum following PS-MP exposure. **(B)** PS-MP-associated fluorescence signal. **(C)** Hematoxylin and eosin (H&E) sections of jejunum and ileum. Scale bar: 200 μm. **(D)** Villus height, crypt depth, and villus height/crypt depth ratio of jejunum and ileum. Data are expressed as the mean ± SD, *n* = 6. Different lowercase letters indicate significant differences (*p* ≤ 0.05), while the same letters indicate no significant differences (*p* > 0.05).

We then investigated the effects of PS-MPs on the development of the jejunum and ileum (Figure 1C). The results of H&E staining showed that the 0.1 mg/d PS-MPs treatment significantly reduced the villus height and the villus height/crypt depth ratio of the jejunum in Chahua chicken No. 2 (*p* < 0.05, Figure 1D). The villus height and villus height/crypt depth ratio of jejunum in the 0.5 mg/d PS-MPs treatment group were significantly lower than those in the CON group (*p* < 0.05, Figure 1D), and the crypt depth of jejunum in the 0.5 mg/d PS-MPs treatment group were significantly greater than those in the CON group (*p* < 0.05, Figure 1D). The 0.05 mg/d PS-MPs treatment significantly reduced the ileal villus height/crypt depth ratio in Chahua chicken No. 2 (*p* < 0.05, Figure 1D). The 0.1 mg/d PS-MPs treatment significantly decreased ileal villus height and increased crypt depth, thereby lowering the villus height/crypt depth ratio (*p* < 0.05, Figure 1D). Similarly, the 0.5 mg/d PS-MPs treatment markedly reduced ileal villus height as well as the villus height/crypt depth ratio (*p* < 0.05, Figure 1D). These results demonstrate that exposure to PS-MPs significantly impairs the intestinal morphology of the jejunum and ileum in Chahua chicken No. 2.

### 3.4 Expression of intestinal barrier-related and inflammatory genes

In Chahua chicken No. 2, exposure to 0.1 and 0.5 mg/d PS-MPs significantly reduced the relative expression level of *ZO-1* and *Claudin-1* in the jejunum of Chahua chicken No. 2 (*p* < 0.05, Figure 2A). In the ileum, 0.05 mg/d PS-MPs significantly downregulated *ZO-1* and *IgA* expression of Chahua chicken No. 2 (*p* < 0.05, Figure 2A), while having no significant effects on *Occludin* and *Muc2* (*p* > 0.05, Figure 2A). Exposure to 0.5 mg/d PS-MPs reduced the expression of *ZO-1*, *Claudin-1*, *Occludin*, *Muc2,* and *IgA* in the ileum of Chahua chicken No. 2 (*p* < 0.05, Figure 2A). Collectively, these results demonstrate that PS-MPs exposure altered the expression of genes associated with intestinal barrier integrity in Chahua chicken No. 2.

**Figure 2:**
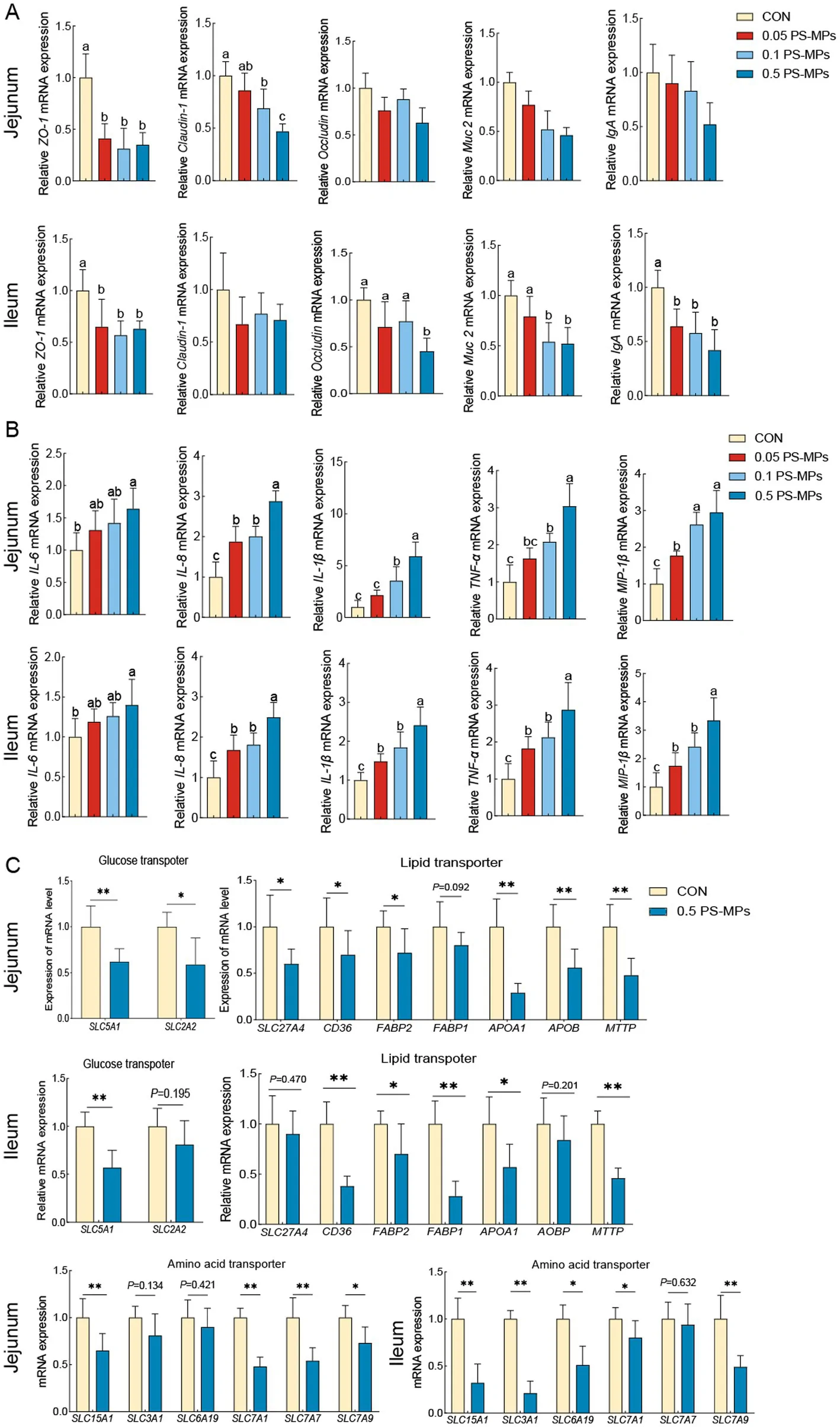
Intestinal barrier function and inflammation. **(A)** Relative mRNA expression of *ZO-1*, *Claudin-1*, *Occludin*, *Muc2*, and *IgA* in jejunum and ileum. Data are expressed as the mean ± SD, *n* = 6. **(B)** Relative mRNA expression of *IL-6*, *IL-8*, *IL-1β*, *TNF-α*, and *MIP-1β* in jejunum and ileum. Data are expressed as the mean ± SD, *n* = 6. **(C)** Relative mRNA expression of glucose, lipid, and amino acid transporter related genes in jejunum and ileum. Data are expressed as the mean ± SD, *n* = 6. * means *P* ≤ 0.05, ** means *P* ≤ 0.01. Different lowercase letters indicate significant differences (*p* ≤ 0.05), while the same letters indicate no significant differences (*p* > 0.05).

Regarding inflammatory markers, 0.05 and 0.1 mg/d PS-MPs significantly upregulated the relative expression of *IL-8* and *MIP-1β* in the jejunum and ileum of Chahua chicken No. 2 (*p* < 0.05, Figure 2B). Treatment with 0.5 mg/d PS-MPs further increased the relative expression of *IL-6*, *IL-8*, *IL-1β*, *TNF-α*, and *MIP-1β* in both the jejunum and ileum of Chahua chicken No. 2 (*p* < 0.05, Figure 2B).

### 3.5 Expression of nutrient transport-related genes

To investigate whether PS-MPs exposure was associated with changes in the expression of genes involved in intestinal nutrient transport, we examined genes related to glucose, lipid, and amino acid transport. The results showed that 0.5 mg/d PS-MPs exposure significantly downregulated the relative expression of glucose transport related genes (*SLC5A1* and *SLC2A2*) and lipid transport related genes (*SLC27A4*, *CD36*, *FABP2*, *APOA1*, *APOB*, and *MTTP*) in the jejunum of Chahua chicken No. 2 (*p* < 0.05, Figure 2C). Similarly, in the ileum, 0.5 mg/d PS-MPs significantly downregulated the relative expression of glucose transport genes *SLC5A1* and lipid transport genes (*CD36*, *FABP2*, *FABP1*, *APOA1*, and *MTTP*) of Chahua chicken No. 2 (*p* < 0.05, Figure 2C). Regarding amino acid absorption and transport, PS-MPs significantly reduced the expression of amino acid transporter-related genes *SLC15A1*, *SLC7A1*, *SLC7A7*, and *SLC7A9* in the jejunum, as well as *SLC15A1*, *SLC3A1*, *SLC6A19*, *SLC7A1*, and *SLC7A9* in the ileum (*p* < 0.05, Figure 2C). These findings indicate that PS-MPs exposure is associated with reduced expression of genes involved in intestinal glucose, lipid, and amino acid transport in Chahua chicken No. 2.

### 3.6 Gut microbiome

Compared with 0.05 PS-MPs group, the Ace, Chao, and Sobs indices of 0.5 PS-MPs were significantly increased in Chahua chicken No. 2 (*p* < 0.05, Figure 3A). Principal coordinate analysis (PCoA) based on the Bray-Curtis distance metric revealed a clear separation among sample groups (*p* < 0.05, Figure 3B). The microbiota composition was evaluated and presented in Figures 3C,D. At the phylum level, the abundance of Bacteroidota in the 0.5 PS-MPs group was significantly higher than that in the 0.05 and 0.1 PS-MPs group, while the abundance of Patescibacteria was significantly higher than that in the CON group (*p* < 0.05, Figure 3E). At the genus level, the abundance of *Lactobacillus* and *Limosilactobacillus* in the 0.05 PS-MPs group was significantly higher than that in the CON and 0.5 PS-MPs group (*p* < 0.05, Figures 3D,F). The abundance of *Anaerobutyricum* and *norank_o__RF39* in the 0.5 PS-MPs group was significantly higher than that in the 0.05 and 0.1 PS-MPs group, while the abundance of *Mediterraneibacter*, *Olsenella*, *norank_o__Clostridia_UCG-014*, and *Faecalitalea* was significantly higher than that in the CON, 0.05 and 0.1 PS-MPs group (*p* < 0.05, Figures 3D,F,G).

**Figure 3:**
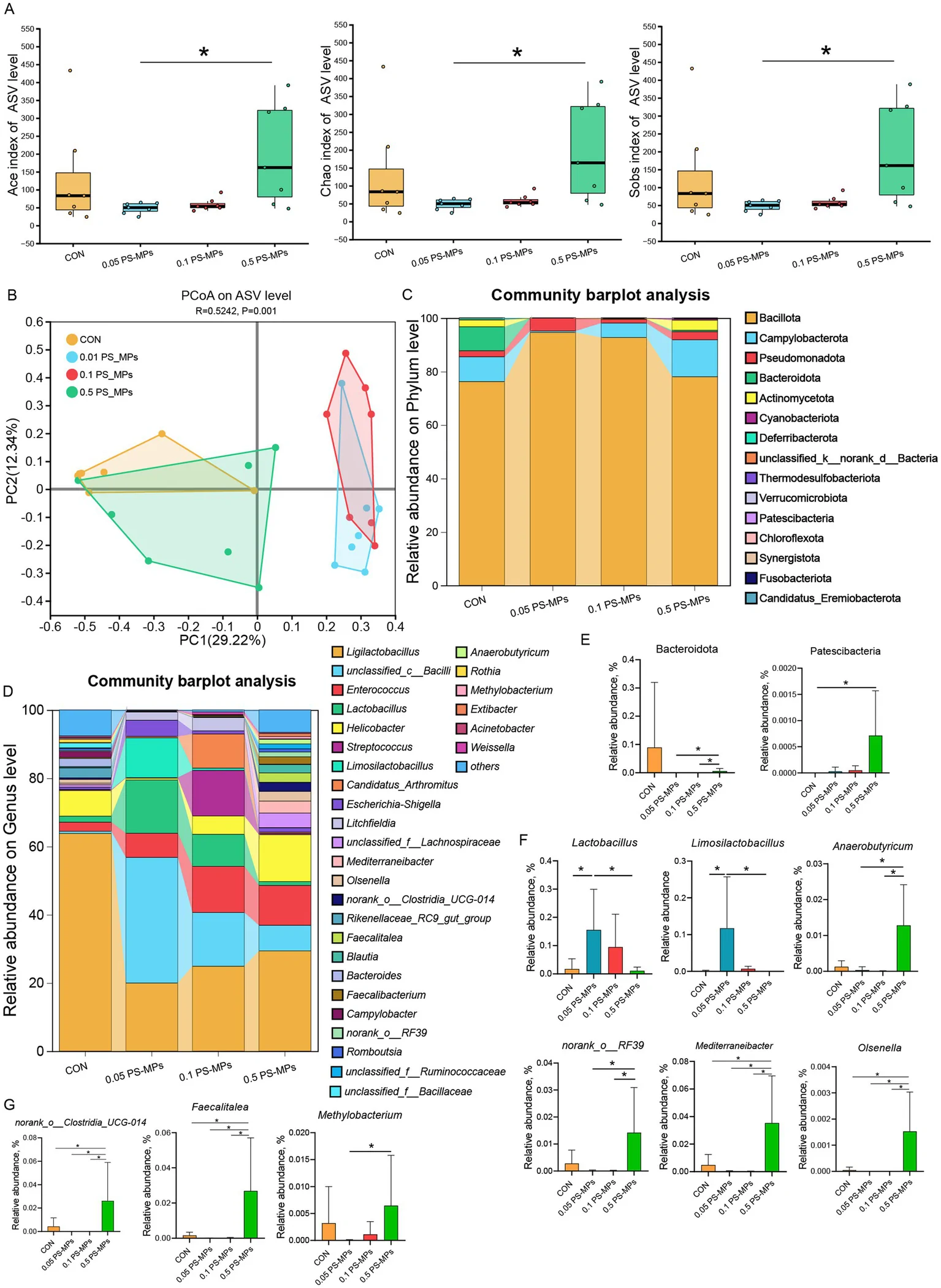
Gut microbiome. **(A)** Alpha-diversity (Ace, Chao, and Sobs indices) of microbiota. **(B)** PCoA analysis of microbiota based on Bray–Curtis distance metrics. **(C)** Relative abundance of microbiota at phylum level, and **(D)** at genus level. **(E)** Exploratory analysis of selected microbial taxa at the phylum level. **(F,G)** Exploratory analysis of selected microbial taxa at the genus level. Less than 1% abundance of phyla or genus was merged into others. Differences among groups were assessed using the Kruskal–Wallis test, followed by Dunn’s multiple comparisons test for pairwise comparisons when appropriate. The microbial taxa presented in panels **(E–G)** were selected based on their relative-abundance patterns and apparent differences among treatment groups and were intended for exploratory comparison rather than an exhaustive differential-abundance analysis. Data are expressed as the mean ± SD, *n* = 7. * means *P* ≤ 0.05.

LEfSe analysis (LDA > 2) showed that *Acinetobacter*, *Christensenellaceae_R-7_group*, *Enorma*, *Acutalibacter*, etc. were significantly enriched in the CON group (Figure 4A); *Limosilactobacillus*, *Pediococcus*, *Lactobacillus*, etc. were significantly enriched in the 0.05 PS-MPs group. *Weissella*, *Litchfieldia*, and *norank_o__Chloroplast* were significantly enriched in the 0.1 PS-MPs group. *Alistipes*, *Anaerobutyricum*, *norank_o__RF39*, *Mediterraneibacter*, *Olsenella*, *norank_o__Clostridia_UCG-014*, and *Faecalitalea*, etc. were significantly enriched in the 0.5 PS-MPs group. Spearman correlation analyses were performed between the 30 most abundant bacterial genera and genes related to intestinal barrier function, inflammation, and nutrient transport. Several positive and negative correlations were observed between bacterial genera and the expression of these genes, but none were statistically significant after Benjamini–Hochberg FDR correction (adjusted *p* > 0.05; Figures 4B,C).

**Figure 4:**
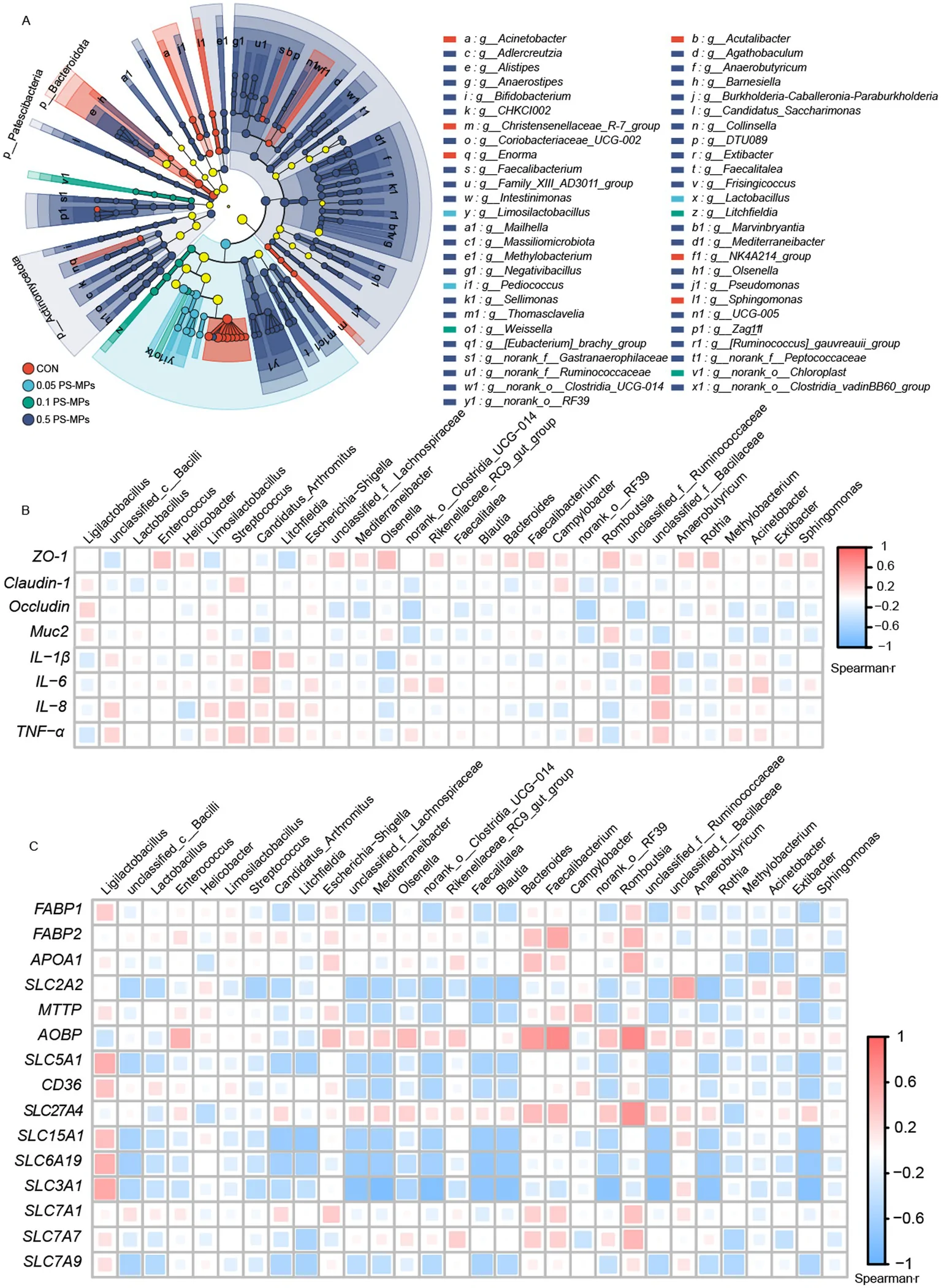
Gut microbiome. **(A)** LEfSe analysis of microbiota (*n* = 7). **(B)** Correlation analysis between intestinal barrier and inflammatory cytokines related genes of ileum and gut microbiota (*n* = 6). **(C)** Exploratory Spearman correlation analysis between genes involved in glucose, lipid, and amino acid absorption and transport of ileum and gut microbiota (*n* = 6). Correlations were evaluated using Spearman’s rank correlation coefficient, and *p*-values were adjusted for multiple comparisons using the Benjamini-Hochberg false discovery rate (FDR) method. * means *p* ≤ 0.05.

## 4 Discussion

Plastics are widely used in daily life and industry due to their convenience (Symeonides et al., 2024). Increasing evidence has suggested that MPs exposure endangers the health of humans and animals (Altunışık, 2023; Cui et al., 2025). In this study, we investigated the distribution of PS-MP-associated fluorescence in the intestines of Chahua chicken No. 2 and evaluated its relationship with growth performance and intestinal health. The results showed that exposure to 0.5 mg/d of PS-MPs significantly reduced body weight, ADG, ADFI, semi-eviscerated yield, eviscerated yield, breast muscle weight, and thigh muscle weight, while increasing FCR in Chahua chicken No. 2. Moreover, 0.5 mg/d PS-MPs exposure was associated with alterations in intestinal morphology, reduced expression of barrier-related genes and nutrient transporter genes, increased expression of inflammatory cytokine genes, and changes in gut microbial community composition.

Studies have reported that ingested MPs can accumulate in the intestines of mice and zebrafish (Qiao et al., 2019; Deng et al., 2018). Similarly, the present study found that the PS-MP-associated fluorescence signal in the jejunum and ileum of chickens increased in a dose-dependent manner. The intestinal effects of MPs are increasingly documented (Xie et al., 2023; Woh et al., 2024). For example, PS-MPs cause intestinal villi damage, mucosal disruption, and reduced mucus secretion in mice (Li et al., 2024a). In weaned piglets, MPs exposure reduced jejunal villus height and downregulates *ZO-1* and *Occludin* expression (Zou et al., 2024). Ducks exposed to MPs also show villus detachment, increased permeability, and decreased *ZO-1* and *Occludin* (Liu et al., 2023). This study further demonstrated that PS-MPs reduced villus height and villus height/crypt depth ratio, and altered the expression of genes associated with tight-junction integrity (*ZO-1*, and *Occludin*) and mucus-related components (*Muc2* and *IgA*) in ileum. These findings suggest that PS-MPs exposure may affect intestinal structural integrity and the expression of genes involved in epithelial barrier maintenance. Since intestinal villi are crucial for nutrient absorption, their shortening likely reduces absorptive surface area (Moor et al., 2018), which may be associated with the observed reduction in body weight, although this relationship was not directly tested in the present study. Upon entering the intestine, MPs can adhere to the mucus layer and directly contact epithelial cells, thereby triggering intestinal inflammation (Woh et al., 2024). MPs-induced intestinal inflammation has been confirmed in mice and poultry (Agrawal et al., 2024; Xu et al., 2023). It is reported that exposure to 5 μm PS-MPs induces liver inflammation and the formation of macrophage extracellular traps (Yin et al., 2023). Consistently, exposure to 0.05 and 0.1 mg/d PS-MPs significantly upregulated *IL-8* and *MIP-1β*, while 0.5 mg/d PS-MPs significantly increased the expression of *IL-6*, *IL-8*, *IL-1β*, *TNF-α*, and *MIP-1β*, supporting a potential pro-inflammatory response to PS-MPs exposure. Long-term MPs retention may affect intestinal processes involved in nutrient digestion and transport. *In vitro* simulated experiments have confirmed that MPs affected lipids and starch digestion (O’Brien et al., 2021; Tan et al., 2020). For example, PS-MPs exposure decreased amylase activity in mussels and alter energy metabolism gene expression (Détrée and Gallardo-Escárate, 2017). In glucose absorption, *SLC5A1* on the apical membrane of intestinal epithelial cells mediates glucose uptake from the intestinal lumen, whereas basolateral *SLC2A2* transports glucose into the bloodstream (Blanco et al., 2017). Studies on zebrafish have found that MP exposure suppress *SLC2A2* expression and reduce glucose uptake (Kim et al., 2025), while in pond loaches, MPs reduced *SLC5A1* expression (Loganathan et al., 2025). However, systematic research on MP effects on nutrient transporters in livestock and poultry remains limited. This study revealed for the first time that PS-MPs exposure significantly reduced *SLC5A1* and *SLC2A2* expression of Chahua chicken No. 2, suggesting altered regulation of genes involved in intestinal glucose transport. This downregulation may also reflect epithelial cells damage, as loss of transporter expression often accompanies structural disruption (Tang et al., 2024). In lipid metabolism, various MPs have been confirmed to inhibit lipid digestion *in vitro* (Kaseke et al., 2023). PS-MPs inhibited lipase activity in simulated human gastrointestinal systems, thereby reducing lipid digestion rates and bioavailability (Tan et al., 2020). Studies on zebrafish indicated that MPs exposure induced oxidative stress, suppress lipid activity and bile acid secretion, and downregulated lipid synthesis and transport genes *CD36*, *DGAT1A*, *DGAT2*, and *MTTP*, ultimately impairing lipid absorption and growth (Zhang et al., 2022). Normally, long-chain fatty acids enter enterocytes via *CD36* and *SLC27A4* (Ko et al., 2020; Chmurzyńska, 2006), are shuttled by *FABP1*/*FABP2*, re-esterified into triglycerides, and assembled with apolipoproteins (*APOB*, *APOA1*) via *MTTP* to form chylomicrons for systemic release (Ko et al., 2020). Here, high-dose PS-MPs significantly downregulated *SLC27A4*, *CD36*, *FABP1*, *APOA1*, *APOB*, and *MTTP* in Chahua chicken No. 2, indicating altered expression of genes involved in fatty acid uptake, intracellular lipid transport, and lipoprotein assembly. These transcriptional changes are consistent with previous observations in zebrafish (Zhang et al., 2022), but their functional consequences for intestinal lipid absorption remain to be established. In addition, MPs also affected amino acid absorption. In zebrafish, PS-MP exposure disturbed amino acid metabolism and alter intestinal amino acid levels (Qiao et al., 2019). Similarly, this study found that PS-MPs downregulated multiple amino acid transporter genes (*SLC15A1*, *SLC3A1*, *SLC6A19*, *SLC7A1*, *SLC7A7*, and *SLC7A9*) in Chahua chicken No. 2, indicating altered expression of genes involved in amino acid transport. These changes may be associated with the reduced growth performance observed following PS-MP exposure, although the present study did not directly measure amino acid absorption or utilization.

The gut microbiota plays a central role in maintaining intestinal epithelial barrier integrity as well as in nutrient digestion and absorption (Wang Y. et al., 2024; Yang et al., 2024). In this study, we observed that exposure to PS-MPs significantly disrupts the gut microbial structure in Chahua chicken No. 2. At the genus level, exposure to 0.5 mg/d PS-MPs was associated with enrichment of *Faecalitalea* and *norank_o__Clostridia_UCG-014*, that have been associated with butyrate production in previous studies (Martín et al., 2023; Wang H. et al., 2024). Meanwhile, the abundance of *Alistipes* and *Faecalitalea*, that were often associated with inflammatory and disease states, was also elevated (Parker et al., 2020). These changes may reflect an alteration of the gut microbial community in response to PS-MP exposure. Although some of the enriched taxa have been associated with butyrate production or other intestinal functions in previous studies, the functional significance of their altered abundance in the present study remains uncertain (Li et al., 2024a). Furthermore, 0.5 mg/d PS-MPs exposure increased the abundance of *Mediterraneibacter*, a genus that has been associated with intestinal mucin degradation in previous studies (Tiffany et al., 2026). Whether this functional activity was altered in the present study remains unknown. Interestingly, 0.05 mg/d PS-MPs exposure increased the abundance of the potential probiotic *Lactobacillus*, which has been reported to alleviate colitis and enhance intestinal barrier function (Wang et al., 2025). However, whether the observed increase in *Lactobacillus* represents an adaptive response to PS-MP exposure or contributes to intestinal homeostasis cannot be determined from the present 16S rRNA sequencing data. Overall, PS-MP exposure was associated with changes in the composition of the gut microbiota, including alterations in taxa previously linked to intestinal homeostasis and inflammatory conditions. Because 16S rRNA sequencing does not directly provide information on microbial functional activity or metabolite production, further metagenomic, metabolomic, and functional studies are needed to determine whether the observed microbial alterations have functional consequences for intestinal barrier integrity, inflammation, nutrient absorption, or growth performance.

## 5 Conclusion

In conclusion, 0.5 mg/d PS-MPs significantly reduced growth performance and was associated with alterations in intestinal morphology, the expression of genes related to epithelial barrier integrity and nutrient transport, inflammatory responses, and gut microbiota composition in Chahua chicken No. 2. Lower doses did not significantly affect growth performance but were associated with changes in barrier-related and inflammatory gene expression and ileal microbiota. These findings highlight the need for strategies to reduce microplastic risks in poultry production. We acknowledge that the use of pristine, spherical 5 μm PS-MPs limits extrapolation to environmental exposures, and that direct functional validation of barrier permeability and nutrient absorption was not performed. Further studies with aged microplastics and functional assays are needed to confirm our findings.

## Data Availability

The original contributions presented in the study are included in the article. All raw sequences are deposited in the NCBI SRA (https://www.ncbi.nlm.nih.gov/sra) under Bioproject accession PRJNA1441598. Further inquiries can be directed to the corresponding author.

## Footnotes

1
www.i-sanger.com
